# Association of *TGFB* -509C>T promoter polymorphism with primary angle closure glaucoma in a North Indian Punjabi cohort

**DOI:** 10.1186/s12886-021-01924-4

**Published:** 2021-04-08

**Authors:** Nanamika Thakur, Rajeev Kumar Pandey, Rashim Mannan, Archna Pruthi, Sanjana Mehrotra

**Affiliations:** 1 Department of Human Genetics, Guru Nanak Dev University, Amritsar, Punjab India; 2 Thermo Fisher Scientific, Bengaluru, Karnataka India; 3 All India Institute of Medical Sciences, New Delhi, India

**Keywords:** Primary glaucoma, Case-control study, Genetic association study, *TGFB* -509C > T, Polymorphism, Neurodegeneration, PACG

## Abstract

**Purpose:**

*Transforming growth factor beta (TGFB)* is an important candidate gene implicated in glaucoma pathogenesis because it affects retinal ganglionic cell survival. The present study assessed the genetic association of -509C > T variant in the *TGFB* promoter region with primary open angle glaucoma (POAG) and primary angle closure glaucoma (PACG) in a North Indian Punjabi population.

**Method:**

A total of 867 subjects (307 POAG, 133 PACG cases and 427 controls) were recruited from the targeted population. Genotyping was done by PCR-RFLP method and the data was analyzed using PLINK software (v1.07). Logistic regression under different genetic models was applied and genotype phenotype correlation was assessed by one-way ANOVA.

**Result:**

A statistically significant difference in the frequency of heterozygotes among PACG cases (53.16%) and controls (30.07%) (***p*** **= 0.0002)** was observed. Genetic model analysis revealed that mutant “TT” genotype conferred 2-fold risk towards PACG development under recessive model (***p*** **= 0.0019)** while dominant model and co-dominant model provided 0.62 and 0.37 fold protection against PACG (***p*** **= 0.025** and ***p*** **= 0.0001**, respectively). Data segregation based on sex revealed a strong protective effect of heterozygous ‘CT’ genotype against progression of PACG among females (***p*** **= 0.002**, OR = 0.37, 95% CI = 0.19–0.70), but conferred 2.14-fold risk among female POAG subjects (***p*** **= 0.013)**.

**Conclusion:**

The study revealed a strong genetic association of -509C > T variant in *TGFB* with PACG in females. There is a need to replicate the results in a larger PACG cohort in other populations and further assess the contribution of sex specific factors in modifying genetic susceptibility to PACG.

## Introduction

Glaucoma is a group of optic neuropathies with multifactorial etiology [1]. Being the second leading cause of blindness worldwide, it is a serious health condition with high socio-economic impact. Its prevalence is expected to rise to 111 million by 2040 with primary open angle (POAG) and angle closure glaucoma (PACG) contributing to bulk of the cases [2]. Common risk factors for the disease include advanced age, elevated intraocular pressure (IOP), high refractive errors (± > 3.00D), decreased corneal thickness, race/ethnicity, and familial history [1, 3]. The adverse visual outcome which is a major concern for glaucoma patients occurs due to gradual degeneration of retinal ganglion cells (RGCs). Sufficient evidence exist to suggest the involvement of chronic neuroinflammation due to altered levels of cytokines under glaucomatous stress conditions in contributing to RGCs death [4, 5]. Among the various cytokines implicated in the pathogenesis of glaucoma, transforming growth factor beta (TGFB) is significant because of its multifaceted role in the ocular milieu [6] specifically in affecting IOP dynamics. Several ex-vivo and in-vitro studies have established that high TGFB level is one causative factor for elevated IOP and patients with increased levels of this cytokine in the aqueous humor (AH) are at a higher risk to develop elevated IOP [7–9]. In one such study by Bhattacharya et al., infusion of TGFB-2 (an isoform of TGFB) to monkey and pig organ-cultured anterior segments led to elevated IOP [10]. An increased IOP was also observed after adenoviral vector mediated active TGFB-2 transfer in rodents [7]. TGFB also mediate excess extracellular (ECM) deposition to induce fibrosis [11] and since in glaucoma extensive ECM remodeling occurs in trabecular meshwork (TM) and ocular nerve head (ONH), dysfunctional TGFB signaling has been associated with glaucoma pathology [8, 12]. In cultured human TM cells, exogenous TGFB treatment may increase the production of proteoglycans and other ECM proteins in these cells, which further reduce the outflow facility of the anterior chamber and thus contribute to increased IOP in the eye of glaucoma patients [13]. TGFB is also involved in the formation of scar tissue in glaucoma via eliciting production of matrix metallopeptidases (MMP)-2 [14]. High levels of TGFβ1 and TGFβ2 isoforms have been reported in different eye fluids including AH and vitreous humor (VH) of glaucoma patients [6, 15]. High intensity fluorescence was detected in immunohistochemistry assay of ONH samples for the receptors of TGFβ-1 isoform in glaucomatous eye [16]. Thus, there is a strong evidence for the possible involvement of TGFβ in glaucoma pathology. Additionally, the promoter and exonic SNPs appear to have an impact on the expression level of *TGFB* and may contribute to inter-individual differences in TGFB levels and hence modify genetic susceptibility to glaucoma [17, 18]. -509C > T promoter variant in *TGFB1* is one such variant which alters its expression [17, 19]. The mutant allele ‘T’ at this site has been associated with increased TGFB-1 expression [17, 20, 21]. Therefore, in the present study, association of the *TGFB-1* -509C > T promoter polymorphism (rs1800469) with primary glaucoma (POAG/PACG) was investigated in a North Indian Punjabi cohort.

## Materials and methods

### Sampling and DNA isolation

Study participants were recruited from Baba Deep Singh Eye hospital, Amritsar (Punjab), India after obtaining written informed consent. The study was approved by the Research Ethical Committee of Guru Nanak Dev University, Amritsar (Institutional Ethical certificate approval number: No. 702/HG/28-06-2019), and the study protocols were in accordance to the principles of Declaration of Helsinki. 440 primary glaucoma patients (POAG = 307 and PACG = 133) were enrolled. For POAG patients, the inclusion was based on following criteria: IOP of greater than 21 mmHg in either of the eyes tested using Goldmann Applanation Tonometry, glaucomatous ONH damage defined as a vertical cup disc ratio (VCDR) 0.7 or greater as adjudged clinically on slit lamp biomicroscopy using hand held + 90 D. This was confirmed using contrast enhanced fundus photograph on optical coherence tomography (OCT) as well as optic disc analysis or glaucomatous visual field defect as detected on automated perimeter using Humpherys Visual Field Analyser using Swedish Interactive Thresholding Algorithm (SITA) standard protocols. PACG cases were also recruited on the basis of above-described criteria along with the presence of at least 180 degrees of closed angle in which the TM is not visible on gonioscopy. The control data set consisted of 427 unrelated age and gender matched subjects without any family history of glaucoma. The controls were all examined, prior to cataract surgery, for IOP less than 21 mmHg, normal visual field, normal optic nerve heads with CDR of *<* 0.5. Individuals with known chronic systemic inflammatory diabetes (random blood sugar-140 mg/Dl; according to American Diabetes Association (ADA) guidelines), autoimmune or immunosuppressive disease as well as a pre-existing ocular disease (diabetic retinopathy, age-related macular degeneration) were excluded from the study. Individuals having ocular hypertension (OHT) were also excluded from the study group. 3 ml of blood sample was collected by venipuncture by a sterile 5 ml of syringe, in a vacutainer containing 0.5 M of EDTA, from all study participants from a local eye hospital after a complete ophthalmic examination. After collection, the blood samples were transferred to the lab in an insulating container containing icepacks and storage was done at − 20 °C for further use.

Genomic DNA was then extracted by using phenol chloroform method [22]. All samples were diluted to 50 ng/μl concentration. Quantification of extracted DNA was done using NanoDrop ND-2000 spectrophotometer (NanoDrop Technologies, Wilmington, DE, USA).

### PCR amplification, Genotyping and Electrophoresis

**Fig. 1:**
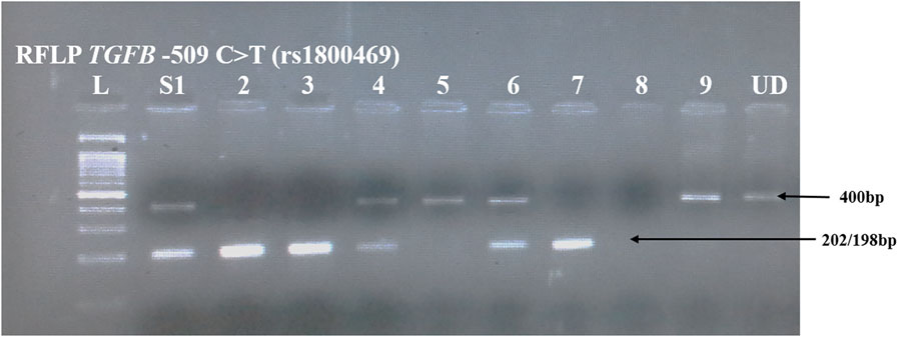
Agarose gel (2.0%) picture depicting the RFLP digested products of *TGFB* -509C > T (rs1800469) promoter polymorphism. Lane 1 consists of 100 bp molecular ladder (L); S2, S3 and S7 represents homozygous CC (198/202 bp); S1, S4 and S6 indicates heterozygous CT (400/198/202 bp) genotypes; S9 represents homozygous TT (400 bp). S8 was a failure. Last well consist of undigested PCR product (UD) of 400 bp

The target promoter flanking region of *TGFB* -509C > T of 400 bp was amplified with the primer sequences FP-5′ CCAGCTAAGGCATGGCACCG 3′ and RP 5′ GCGGTGTGGGTCACCAGAGA 3′ designed by Primer 3 software. The PCR was performed in a final reaction volume of 15 μl containing 50 ng DNA template, 1X Taq Polymerase buffer reagent, 2.5 mmol of each deoxynucleotide triphosphate (dNTPs) (3B BlackBio Biotech India Ltd), 20 mmol MgCl_2_, 0.3 μmol/μl of each primer, and 0.09 units Taq DNA polymerase (3B BlackBio Biotech India Ltd.; 5 U/μl). The cycling conditions were: initial denaturation at 95 °C for 10 min, followed by 35 cycles at 95 °C for 45 s, 65 °C for 45 s and 72 °C for 45 s, with a final extension at 72 °C for 10 min. After amplification, the PCR products were digested with 3 units of Bsu-36I (New England Biolabs) for 12 h at 37 °C. The digested products were separated by electrophoresis. Briefly 2.5 g of agarose (Sigma-Aldrich) was dissolved in 100 ml of 1X TBE buffer (pH 8.0) by boiling the solution. 5 μl of ethidium bromide (10 mg/ml) (GeNei™) was added after allowing the solution to cool to 60 °C. The gels were allowed to solidify and placed in the horizontal electrophoresis chamber containing 1X TBE buffer (pH 8.0). 3 μl of each PCR product and 2 μl of gel loading dye were mixed and loaded in each well. For RFLP, 10 μl of digested products mixed with 5 μl gel loading dye were loaded in each well. The appropriate molecular weight marker was loaded in parallel to compare the sizes of PCR products and RFLP digested amplicon. Electrophoresis was carried out at 100 V for 30 min and the products were visualized under UV transilluminator and photographed. The genotypes were scored on the basis of Restriction Fragment Length Polymorphism (RFLP) pattern as given in Fig. 1.

### Statistical analysis

Descriptive statistical analysis for demographic and clinical characteristics for study participants was performed using GraphPad Prism 5.0. The results were tabulated as mean ± standard deviation (SD). Association analyses were performed using PLINK software (v1.07). Odds ratios (ORs) for various genetic models, dominant, recessive, additive and allelic were calculated. *P*-values of less than 0.05 were considered to be statistically significant. To investigate the association of the variant with IOP and VCDR which are known endophenotypes of glaucoma, the values for right eye was chosen arbitrarily (as the mean for both eyes was same, Table 1) for ANOVA. Binary logistic regression was applied for correcting possible confounders by using SPSS Software Version 21 [23].

**Table 1:** Baseline demographic and clinical parameters among cases and controls

Factors	Cases (Mean ± SD)	Controls (Mean ± SD)	p-value
Age	59.92 ± 12.81	59.46 ± 11.56	0.573
CD Right eye	0.72 ± 0.39	0.23 ± 0.08	0.000*
CD Left eye	0.73 ± 0.39	0.25 ± 0.09	0.000*
IOP Right eye	22.41 ± 8.89	14.09 ± 3.51	0.000*
IOP Left eye	22.90 ± 9.89	14.24 ± 3.43	0.000*

*p* value < 0.05^*^ was considered to be statistically significant

## Results

### Demographic and clinical characteristics of the study participants

**Fig. 2:**
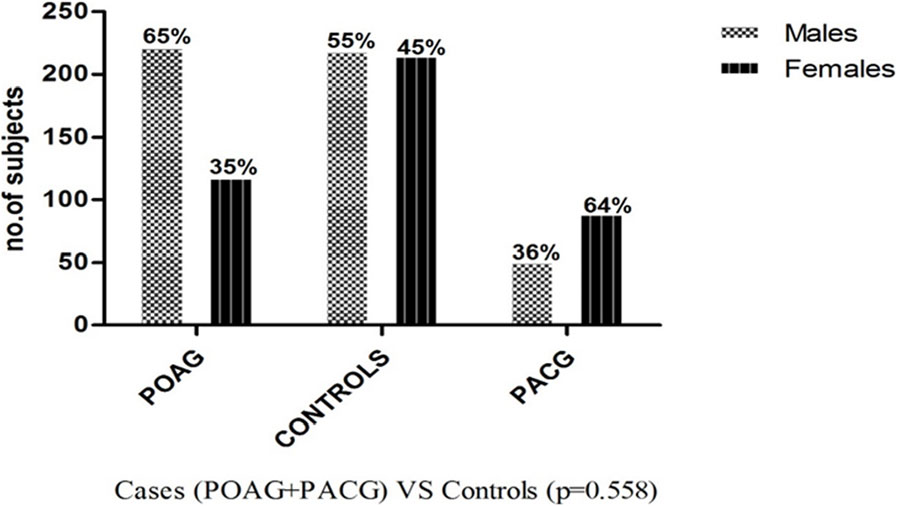
Frequency distribution of males and females among POAG and PACG cases with respect to control subjects

The frequency distribution of demographic factors, sex and age among patients and control subjects is shown in Table 1 and Fig. 2. The mean age among primary glaucoma cases was 59.92 years with standard deviation (SD) of ±12.81, while the mean age among controls was 59.46 with SD of ±11.56. Males had higher prevalence of POAG (65%) while females had a higher prevalence of PACG (64%) in accordance with several other epidemiological surveys where a higher prevalence of POAG has been reported in males while females are at a higher risk for PACG [24]. Total number of males and females in cases (both POAG/PACG) and controls did not show any significant difference (*p* = 0.558) as given in Fig. 2.

These results highlight the gender difference in glaucoma prevalence; women are known to be at a higher risk for PACG [24]. The mean CDR for right and left eye in cases was 0.72 and 0.73, respectively, which was higher with respect to control subjects (0.23 and 0.25) for both right and left eyes, respectively. The mean IOP in both eyes was higher in cases as compared to controls (Table 1) as per the inclusion criteria for recruitment of patients.

### -509C > T *TGFB* polymorphism revealed a borderline significance with POAG

The observed genotype count for -509C > T variant followed Hardy Weinberg equilibrium (HWE) in controls (*p* = 0.144). No significant difference was observed in the genotype frequency (CC, CT and TT genotypes) of combined (28.41, 51.36 and 20.23%) and POAG cases (24.10, 60.59 and 15.31%) with respect to controls (28.12, 53.16 and 18.74%) (Table 2). The frequency of ‘C’ and ‘T’ allele was 54.09 and 45.91% among combined cases, 54.40 and 45.60% in POAG patients respectively, while controls had 54.69% of major allele ‘C’ and 45.31% of minor allele ‘T’. This difference was not significant among any of the two groups; *p* = 0.804 and *p* = 0.913 for combined and POAG cases, respectively with respect to control subjects. Genetic model analysis revealed a borderline significant association (***p*** **= 0.045**) of this variant under dominant model, however the results were not significant after applying corrections for confounders like age, sex and family history (p_corr_ = 0.181).

**Table 2:** Distribution of allele/genotype frequencies and genetic model analysis for *TGFB* − 509C > T (rs1800469) among all cases, POAG/PACG subtypes and controls

SNP rs1800469				
Genotype/Allele	All Cases ***n*** = 440(%)	Controls ***n*** = 427(%)	p-value	OR (95% CI)
C	476 (54.09)	467 (54.69)	**Ref**	
T	404 (45.91)	387 (45.31)	0.804	1.02 (0.84–1.23)
CC	125 (28.41)	120 (28.10)	**Ref**	
CT	226 (51.36)	227 (53.16)	0.775	0.95 (0.70–1.30)
TT	89 (20.23)	80 (18.74)	0.742	1.06 (0.72–1.58)
CT + TT > CC Dominant model	315 > 125	307 > 120	0.920 #0.950	0.98 (0.73–1.32)
				1.01 (0.73–1.39)
TT > CT + CC Recessive model	89 > 351	80 > 347	0.579 #0.196	1.09 (0.78–1.53)
				1.26 (0.88–1.81)
CT > TT + CC Co-dominant	226 > 214	227 > 200	0.596 #0.326	0.93 (0.71–1.21) 0.86 (0.64–1.15)
	**POAG** ***n*** **= 307 (%)**	**Controls n = 427(%)**		
C	334 (54.40)	467 (54.69)	**Ref**	
T	280 (45.60)	387 (45.31)	0.913	1.01 (0.82–1.24)
CC	74 (24.10)	120 (28.10)	**Ref**	
CT	186 (60.59)	227 (53.16)	0.110	1.32 (0.93–1.88)
TT	47 (15.31)	80 (18.74)	0.837	0.95 (0.60–1.51)
CT + TT > CC Dominant model	233 > 74	307 > 120	0.225 #0.267	1.23 (0.87–1.72)
				1.23 (0.85–1.79)
TT > CT + CC Recessive model	47 > 260	80 > 347	0.226 #0.647	0.78 (0.52–1.16)
				0.90 (0.59–1.38)
CT > TT + CC Co-dominant	186 > 121	227 > 200	0.045* #0.181	1.35 (1.00–1.82)
				1.25 (0.90–1.73)
**rs1800469**	**PACG** ***n*** **= 133(%)**	**Controls n = 427(%)**	**p-value**	**OR (95% CI)**
C	142 (53.38)	467 (54.69)	**Ref**	
T	124 (46.62)	387 (45.31)	0.710	1.05 (0.79–1.38)
CC	51 (38.35)	120 (28.10)	**Ref**	
CT	40 (30.07)	227 (53.16)	0.0002*	0.41 (0.25–0.66)
TT	42 (31.58)	80 (18.74)	0.404	1.23 (0.75–2.03)
CT + TT > CC Dominant model	82 > 51	307 > 120	0.025* #0.087	0.62 (0.41–0.94)
				0.66 (0.41–1.06)
TT > CT + CC Recessive model	42 > 91	80 > 347	0.0019* #0.001*	2.00 (1.29–3.10)
				2.30 (1.39–3.79)
CT > TT + CC Co-dominant	40 > 93	227 > 200	< 0.0001* # < 0.0001*	0.37 (0.24–0.57)
				0.35 (0.22–0.57)

* Indicates significant *p*-value ≤0.05; OR = Odds Ratio, CI = Confidence Interval

# indicates statistically significant *p*-value and OR at 95% CI, after applying correction for confounding variables

### -509C > T *TGFB* polymorphism revealed significant association with PACG

The allele and genotype frequency distribution along with genetic model analysis for PACG cases and controls are given in Table 2. Although no significant difference in allele frequency was observed among PACG cases and controls, the heterozygous CT genotype was overrepresented in controls (53.16%) as compared to cases (30.07%) (***p*** **= 0.0002**). Analysis of genotypic distribution revealed 2 folds increased susceptibility towards PACG development under recessive genetic model (TT > CT + CC) (***p*** **= 0.0019**, OR = 2.00; 95% CI = 1.29–3.10) which increased to 2.30 folds after applying binary logistic regression for possible confounders (**p**_**corr**_ **= 0.001**, OR = 2.30; 95% CI = 1.39–3.79). Dominant (CT + TT > CC) (***p*** **= 0.025**, OR = 0.62; 95% CI = 0.41–0.94) and co-dominant models (CT > CC + TT) (*p* < 0.0001, OR = 0.37; 95%CI = 0.24–0.57) also revealed a significant association with PACG but this significance after applying corrections for confounding variables was retained only with codominant model and CT genotype conferred 0.35 times higher protection against PACG development (**p**_**corr**_ **< 0.0001**, OR = 0.35; 95% CI = 0.22–0.57) (Table 2).

### Significant association of -509C > T polymorphism with POAG/PACG subgroups in females

The data was stratified based on sex and reanalyzed (Table 3) to assess sex specific contribution to genetic association with -509C > T variant. The difference in frequency of CC, CT and TT genotypes was marginally significant among male PACG cases (37.25, 37.25 and 25.50%) as compared to control males (26.03, 52.97 and 21.00%) (*p* = 0.050, OR_CT_ = 0.49; 95% CI = 0.24–1.00) but not among POAG males (27.23%; 58.41 and 14.36%) (*p* = 0.818, OR_CT_ = 1.05; 95% CI = 0.67–1.65). In females, this difference was significant in both POAG (17.14, 64.76 and 18.10%) (***p*** **= 0.013**, OR = 2.14; 95% CI = 1.17–3.92) and PACG subjects (39.02, 25.61 and 35.37%) (***p*** **= 0.002**, OR = 0.37; 95% CI = 0.19–0.70) as compared to control female subjects (30.29, 53.36 and 16.35%) for CC, CT and TT genotypes respectively. Additionally, a statistically significant association of *TGFB* -509C > T promoter variant was observed with POAG females (**p = 0.013**, OR = 2.10; 95% CI = 1.16–3.77) under dominant (CT + TT > CC) and PACG females (***p*** **= 0.0005**, OR = 2.80; 95% CI = 1.56–5.01) under recessive (TT > CT + CC) genetic model. After applying corrections, this significance was lost under dominant model for POAG females (p_corr_ = 0.125, OR = 1.63; 95% CI = 0.87–3.05), but not among PACG females under recessive model (**p**_**corr**_ **= 0.001**, OR = 2.91; 95% CI = 1.53–5.59).

**Table 3:** Sex wise distribution of allele and genotype frequencies and genetic model analysis for *TGFB* − 509 C > T (rs1800469) among POAG and PACG cases with respect to controls

**Males with POAG and PACG vs. Control male subjects**								
**Allele/Genotype**	**POAG** ***n*** **= 202 (%)**	**Controls** ***n*** **= 219 (%)**	**p-value**	**OR (CI)**	**PACG** ***n*** **= 51 (%)**	**Controls n = 219 (%)**	**p-value**	**OR (CI)**
C	228 (56.44)	230 (52.51)	**Ref**		57 (55.88)	230 (52.51)	**Ref**	
T	176 (43.56)	208 (47.49)	0.253	0.85 (0.65–1.12)	45 (44.12)	208 (47.49)	0.539	0.87 (0.56–1.34)
CC	55 (27.23)	57 (26.03)	**Ref**		19 (37.25)	57 (26.03)	**Ref**	
CT	118 (58.41)	116 (52.97)	0.818	1.05 (0.67–1.65)	19 (37.25)	116 (52.97)	0.050*	0.49 (0.24–1.00)
TT	29 (14.36)	46 (21.00)	0.160	0.65 (0.36–1.18)	13 (25.50)	46 (21.00)	0.687	0.84 (0.37–1.89)
**Genetic Models**								
CT + TT > CC Dominant model	147 > 55	162 > 57	0.780 #0.886	0.94 (0.61–1.44)	32 > 19	162 > 57	0.110 #0.173	0.59 (0.31–1.12)
				1.03 (0.64–1.66)				0.58 (0.26–1.26)
TT > CT + CC Recessive model	29 > 173	46 > 173	0.076 #0.433	0.63 (0.37–1.05)	13 > 38	46 > 173	0.485 #0.253	1.28 (0.63–2.61)
				0.81 (0.47–1.37)				1.60 (0.71–3.61)
**Females with POAG and PACG vs. Control female subjects**								
**Allele/Genotype**	**POAG** ***n*** **= 105 (%)**	**Controls** ***n*** **= 208 (%)**	**p-value**	**OR (CI)**	**PACG** ***n*** **= 82 (%)**	**Controls** **n = 208 (%)**	**p-value**	**OR (CI)**
C	104 (49.52)	237 (56.97)	**Ref**		85 (51.83)	237 (56.97)	**Ref**	
T	106 (50.48)	179 (43.03)	0.077	1.34 (0.96–1.88)	79 (48.17)	179 (43.03)	0.262	1.23 (0.85–1.76)
CC	18 (17.14)	63 (30.29)	**Ref**		32 (39.02)	63 (30.29)	**Ref**	
CT	68 (64.76)	111 (53.36)	0.013*	2.14 (1.17–3.92)	21 (25.61)	111 (53.36)	0.002*	0.37 (0.19–0.70)
TT	19 (18.10)	34 (16.35)	0.086	1.95 (0.90–4.21)	29 (35.37)	34 (16.35)	0.119	1.67 (0.87–3.22)
**Genetic Models**								
CT + TT > CC Dominant model	87 > 18	145 > 63	0.013* **#**0.125	2.10 (1.16–3.77)	50 > 32	145 > 63	0.154 #0.256	0.67 (0.39–1.15)
								0.71 (0.39–1.28)
				1.63 (0.87–3.05)				
TT > CT + CC Recessive model	19 > 86	34 > 174	0.724 #0.806	1.11 (0.60–2.07)	29 > 53	34 > 174	0.0005* #0.001*	2.80 (1.56–5.01)
				1.09 (0.54–2.20)				2.91 (1.53–5.59)

* Indicates significant *p*-value; OR = Odds Ratio, CI = Confidence Interval

# indicates statistically significant *p*-value and OR at 95% CI, after applying correction for age and family history

### Association of IOP and CDR with *TGFB* -509C > T polymorphism

No significant difference in the mean values of CDR (*p* = 0.563) and IOP (*p* = 0.108) was observed among CC, CT and TT genotypes, although mean value of CDR was higher among heterozygotes (Table 4).

**Table 4:** Comparison of clinical parameters (mean ± SD) in three genotypes of *TGFB* -509 C > T (rs1800469) polymorphism

Genotype (rs1800469)	CDR	IOP
CC (125)	0.72 ± 0.17	22.79 ± 9.00
CT (226)	0.75 ± 0.54	21.68 ± 8.23
TT (89)	0.70 ± 0.15	23.92 ± 9.41
F-value	0.575	2.235
p-value	0.563	0.108

CDR-Cup-to-disc ratio; IOP-Intraocular pressure

*p* < 0.05 were considered to be statistically significant

## Discussion

In the current analysis, association of -509C > T promoter variant in *TGFB1* with primary glaucoma was assessed. In the ocular microenvironment, TGF-B1 is mostly found in the AH, VH and tears [25–27] where it is involved in the production of proteoglycans like chondroitin sulfate. The latter is the main component of the ECM of the TM cells, which alters AH outflow from the eye. Therefore, any alteration in TGFB1 levels may affect the AH outflow facility, and thereby contributing to increase in IOP which is the main risk factor for glaucoma [28, 29]. Studies conducted in the past 20 years have repeatedly demonstrated elevated levels of TGFB in the AH of POAG patients [30–34].

In the present study, a statistically significant difference was observed between the frequency of heterozygotes ‘CT’ among PACG subgroup of patients (53.16%) and controls (30.07%) (***p*** **= 0.0002)**. However, allele frequencies did not differ in combined (*p* = 0.804) as well as two glaucoma subtypes, POAG/PACG cases (*p* = 0.913; *p* = 0.710, respectively). On segregating the data into males and females, the study found a protective effect of heterozygous ‘CT’ genotype against progression of PACG among both males and females (***p*** **= 0.050**, OR = 0.49; 95% CI = 0.24–1.00 and ***p*** **= 0.002**, OR = 0.37; 95% CI = 0.19–0.70, respectively), while the ‘CT’ genotype conferred 2.14-fold risk among female POAG subjects (***p*** **= 0.013)**. Genetic model analysis indicated that the “TT” genotype conferred 2-fold risk towards PACG development under recessive model (***p*** **= 0.0019)** while the combination of CT + TT under dominant model and heterozygous ‘CT’ genotype under co-dominant model provided 0.62 and 0.37 fold protection against PACG (***p*** **= 0.025 a**nd ***p*** **= 0.0001)** respectively. The significance was retained after applying corrections for confounders under recessive (**p**_**corr**_ **= 0.001**, OR = 2.30; 95% CI = 1.39–3.79) and codominant models (**p**_**corr**_ **= 0.0001**, OR = 0.35; 95% CI = 0.22–0.57). For POAG, only marginal significance was observed under co-dominant model (***p*** **= 0.045,** OR = 1.35; 95% CI = 1.00–1.82), which did not survive corrections. The positive association signal between PACG and -509C > T variant was contributed by females as the ‘TT’ genotype under recessive genetic model gave 2.80-fold risk towards PACG development among female subjects (***p*** **= 0.0005**, OR = 2.80; 95% CI = 1.56–5.01; **p**_**corr**_ **= 0.001**, OR = 2.91, 95% CI = 1.53–5.59). From these results, it can be assumed that −509C > T variant modifies genetic susceptibility to PACG in females. Such sex-specific differences in disease susceptibility are also known in cardiovascular and autoimmune diseases and may be attributed to hormonal and epigenetic differences between the sexes [35]. In glaucoma, however the effect of sex in genetic association with SNPs has not been widely investigated and to the best of our knowledge, only a single study determined whether genetic association at the *9p21* locus is influenced by sex in POAG [36]. Further, no study till date has assessed genetic association with SNPs in PACG in sex specific context. The results for female specific association of *TGFB* in PACG in the present study may suggest a plausible crosstalk pathway between estradiol and cytokines signaling. There is sufficient evidence in favor of role of estradiol in pathogenesis of glaucoma and it may have therapeutic potential for POAG in future [37–39]. Among the pleiotropic effects of estrogen, one role has been in modulating expression of anti-inflammatory cytokines like TGFB [40] through which it may alter the cytokine balance in the ocular microenvironment and increase susceptibility to glaucoma. The negative results with POAG in current study are similar to another study conducted on South Indian population (POAG cases = 106 and controls = 104) where no association was reported between *TGFB-1* -509C > T polymorphism and POAG or IOP and CDR [41]. In contrast to these reports, significant association of -509C > T polymorphism was reported with POAG among East Iranian population (POAG cases = 112 and controls = 112) with ‘T’ allele as the risk allele (*p* = 0.005; OR = 1.73). The frequency of heterozygous ‘CT’ genotype (45.6%) was higher among POAG patients as compared to controls (38.4%) (*p* = 0.020; OR = 1.93) [42]. Grainger et al. also observed that the allele ‘T’ at -509 position, confers risk towards POAG progression and led to higher plasma levels of TGFB as compared to those who carried ‘C’ allele at this position [17]. However, lack of inclusion of PACG subcategory, absence of sex-based stratification among POAG and PACG cases despite reports stating sex to be an important risk factor for glaucoma, and insufficient sample size were some limitations of the previous studies. With a robust sample size and sex-based segregation in POAG and PACG performed in the present study, it can be concluded that *TGFB* gene variant is associated with PACG in the North Indian Punjabi population. For − 509 > C variant in *TGFB*, a potential female specific association was observed with PACG risk. The present study provides a strong impetus to conduct additional analyses in other populations to specifically test for potential sex specific effects in the genetic architecture of POAG and PACG.

## Data Availability

The datasets generated and/or analyzed in the North Indian Punjabi glaucomatous population are included in the manuscript. Any additional information is available upon request from the corresponding author.

## Abbreviations

TGFB: Transforming growth factor beta
POAG: Primary Open Angle Glaucoma
PACG: Primary Angle Closure Glaucoma
PCR-RFLP: Polymerase Chain Reaction-Restriction Fragment Length Polymorphism
OR: Odds Ratio
RGCs: Retinal Ganglionic Cells
IOP: Intraocular Pressure
VCDR: Vertical Cup Disc ratio
OCT: Optical Coherence Tomography
SITA: Swedish Interactive Thresholding Algorithm
ADA: American Diabetes Association
OHT: Ocular Hypertension
EDTA: Ethyelene DiamineTetraacetic Acid
SD: Standard Deviation
HWE: Hardy Weinberg Equilibrium
VH: Vitreous Humor
AH: Aqueous Humor
CI: Confidence Interval
